# Plasmidomics: studying plasmids as ecological entities beyond their hosts

**DOI:** 10.1099/mgen.0.001846

**Published:** 2026-10-09

**Authors:** Bradd Mendoza-Guido, Keilor Rojas-Jimenez

**Affiliations:** 1 Instituto de Investigaciones en Salud, Universidad de Costa Rica, San José, Costa Rica; 2 Escuela de Biología, Universidad de Costa Rica, San Pedro de Montes de Oca, Costa Rica

**Keywords:** horizontal gene transfer, metagenomics, microbiology, mobile genetic elements, molecular evolution, molecular symbionts

## Abstract

Plasmids are mobile genetic elements with autonomous replication, whose ecology extends beyond individual bacterial hosts. As molecular symbionts, they have the potential to traverse bacterial taxa and environments, disseminating adaptive genes and shaping microbial community structure through dynamics that are likely decoupled from host taxonomy. Plasmidomics – the omics discipline dedicated to the study of plasmids – has revealed that plasmid diversity and distribution respond to environmental gradients independently of their hosts, underscoring their roles as ecological entities. However, the field faces critical methodological constraints: short-read assemblies fragment plasmid sequences, culture-dependent approaches underrepresent environmental diversity and most metagenomic methods fail to capture plasmid–host associations. In addition, a universally accepted classification framework is still lacking. Advancing plasmidomics will require the integration of long-read sequencing, Hi-C proximity ligation, meta-epigenomics and ecology-informed classification frameworks grounded in genomic and functional criteria. Together, these approaches are essential to uncover the true diversity, evolutionary significance and ecological dynamics of plasmids across complex microbial ecosystems.

**Impact Statement**

Understanding plasmid dynamics is essential for addressing major challenges in microbiology, including the spread of antimicrobial resistance and the adaptation of microbial communities to environmental changes. While plasmid research has traditionally focused on their role in gene transfer within specific hosts, growing evidence supports a broader perspective in which plasmids are considered ecological units operating across microbial communities and environments under their own dynamics. This perspective contributes to the field by synthesizing recent conceptual and methodological advances in plasmidomics and highlighting key gaps that limit our ability to fully characterize plasmid diversity and function. In particular, we emphasize persistent challenges in detecting and reconstructing plasmids from environmental data, as well as in accurately assigning host taxonomy. By framing these limitations within an ecological context, we provide a critical evaluation of current approaches and their implications for interpreting plasmid-driven processes. Importantly, we also highlight the need for integrative strategies that combine complementary approaches to advance towards a standardized, ecology-informed plasmid classification system. This perspective aims to support the development of more robust methodological and conceptual frameworks for studying plasmids, ultimately improving our ability to understand their true role in natural ecosystems.

## Data Summary

No new data were generated in this work.

## Plasmids as ecological entities

Plasmids are mobile genetic elements (MGEs) characterized by dedicated replication systems that enable autonomous replication, often supported by maintenance functions that coordinate replication with the host cell cycle [1]. They have the potential to traverse bacterial taxa and environments, accumulating and disseminating adaptive genes across microbial communities. Beyond their canonical role in horizontal gene transfer (HGT), plasmids exert a direct influence on bacterial ecology and evolution [2, 3]. For instance, plasmid communities can function as dynamic genomic reservoirs in which plasmid-encoded genes evolve under selective regimes distinct from those acting on chromosomes, partly driven by elevated copy numbers and the coexistence of multiple plasmid variants within a single host [2].

Interactions among plasmid populations within the same host support their role as ecological and evolutionary entities, as they can be favoured or purged by evolutionary forces acting at their own level, sometimes independently of processes at the level of the host population [4]. Rossine *et al*. [5] demonstrated that intracellular dynamics directly modulate plasmid competition. Reduced replication variability (e.g. mediated by hemimethylation) can slow genetic drift and prolong plasmid coexistence within cells. In contrast, plasmids with increased transcriptional activity exhibited reduced within-cell fitness, thereby favouring the fixation of less transcriptionally active plasmids. Nonetheless, when a plasmid encodes a beneficial trait (e.g. antibiotic resistance), it may be favoured at the host level and maintained despite incurring an intracellular fitness cost. Under this framework, plasmids carrying alleles with limited ecological relevance are more decoupled from their hosts' ecology and evolutionary dynamics, whereas those with stronger fitness effects at the host level remain more tightly linked [4].

This relationship also varies with host range. Some plasmids exhibit a narrow host range, reflecting constraints on plasmid–host compatibility. Successful establishment in a new host depends on compatibility at multiple stages, including transfer and entry, replication within the host and evasion of host defence systems [6]. When plasmids are restricted to specific hosts, selective pressures acting on the host are more likely to shape plasmid ecology in parallel. In contrast, when plasmids are able to transfer across diverse taxa, their abundance and selection dynamics become more decoupled from any single host’s biology.

Nevertheless, plasmids always depend on a bacterial host cell as the intracellular environment in which they persist and interact with other elements. Therefore, plasmids and other MGEs are best understood as molecular symbionts, engaging in ecological host interactions that span a parasitism–mutualism continuum shaped by evolutionary trajectories, intracellular competition and environmental selective pressures [3, 7, 8]. These forces may drive plasmid diversification in ways that are only partially coupled to host ecology [9, 10].

This perspective provides a framework for interpreting key aspects of plasmid biology beyond the constraints of individual hosts, encompassing technological limitations, challenges in current classification systems and plasmid ecological dynamics. Advancing the field requires integrative plasmidomics approaches that combine genomic, ecological and evolutionary perspectives. In particular, metagenomic strategies incorporating long-read sequencing, proximity-ligation and meta-epigenomic methods are essential to recover complete plasmid architectures and resolve their interactions with host organisms and other molecular symbionts in natural communities, alongside the development of robust and biologically meaningful classification systems. Together, these advances are critical to uncover the true diversity, ecological dynamics and evolutionary significance of plasmids across complex and largely uncultured ecosystems.

## Evolution and challenges of plasmidomics

Plasmidomics is the omics discipline focused on studying plasmids, including their interactions with host organisms, other molecular entities and biotic and abiotic components of their ecosystems. If genomics studies genomes, plasmidomics studies the collection of plasmids (the ‘plasmidome’) present in a cell, organism and environment.

The sequencing of plasmids and bacterial genomes has been widely applied for decades, substantially advancing our understanding of plasmid biology and their roles in bacterial adaptation. In recent years, the rapid expansion in the number of available bacterial genomes (both draft and complete) has driven the development of numerous bioinformatic tools and databases aimed at plasmid identification and classification. Initial methods relied on the identification of hallmark plasmid features, such as replication initiation and relaxase proteins, and the assignment of incompatibility groups through similarity searches against curated databases, as implemented in tools like PlasmidFinder [11].

However, plasmid reconstruction from draft genomes remains inherently difficult, largely due to the abundance of repetitive elements and mobile genetic components that disrupt assembly continuity and lead to fragmented or ambiguous contigs [12]. To overcome these limitations, subsequent approaches incorporated structural information by clustering contigs based on shared genomic distances and incompatibility constraints to infer contigs belonging to the same plasmid, as exemplified by tools such as MOB-suite [13]. In parallel, machine-learning-based (MLB) methods emerged, classifying contigs as plasmid or chromosomal by integrating sequence composition, gene content and architectural features, thereby reducing dependence on complete assemblies and reference bias [14, 15]. These strategies have improved plasmid identification in draft genomes, supported by the biological assumption that bacteria typically harbour a limited number of extrachromosomal replicons and that plasmids of the same incompatibility group rarely coexist.

More recently, these methodological frameworks have been adapted to metagenomic data, where plasmid detection is further complicated by extensive contig fragmentation, high plasmid diversity and the lack of direct host linkage information. In this context, MLB tools specifically trained on metagenomic datasets have become central to plasmid discovery [10, 14, 16]. Notably, Yu *et al*. demonstrated that plasmid sequences predicted from human gut metagenomes using PlasX could be recovered from cultured isolates and experimentally transferred between *Bacteroides fragilis* strains [17]. However, a major limitation in plasmid research remains the poor characterization of uncultured bacterial communities and, consequently, their plasmids.

Currently available metagenome-assembled genome (MAG) reconstruction methods tend to miss the recruitment of plasmid sequences due to differences in G+C content, copy number and coverage relative to chromosomal DNA [18]. This limitation results in a substantial underrepresentation of plasmids from uncultured bacteria and limits the diversity captured in reference datasets. Therefore, MLB models trained on restricted and biased collections of well-characterized plasmids may fail to detect novel or divergent plasmid sequences. Additionally, many proteins are shared between plasmids and other MGE, such as integrative and conjugative elements (ICEs), which can lead to the misclassification of ICE-derived contigs as plasmids and vice versa [19]. In addition, the ability of ICEs to transition into plasmids and of plasmids to integrate into chromosomes as ICEs further complicates their discrimination [20, 21].

Long-read sequencing technologies offer clear advantages for plasmid assembly and detection, particularly in metagenomic datasets. Both PacBio and Oxford Nanopore platforms routinely generate reads exceeding 10 kb, which can span most plasmids, as these are often smaller than this size [1]. For larger plasmids, long reads are frequently sufficient to bridge repetitive regions that otherwise prevent correct overlap and contig reconstruction when using short reads alone. Despite these advantages, applications of long-read sequencing for plasmid identification in metagenomes remain scarce [22–24], likely due to the higher cost and technical complexity of long-read metagenomic sequencing.

Moreover, systematic benchmarking studies comparing plasmid recovery and classification across long-read, short-read and hybrid approaches remain limited. The latter comparison is particularly important, as genome assemblers based on long-read sequencing have been reported to miss small plasmids (<10 kb) [25, 26]. Thus, despite their advantages in resolving plasmid structure, long-read approaches may underestimate plasmid diversity in metagenomic samples.

An alternative strategy involves enriching plasmids from microbial communities prior to sequencing to reduce chromosomal DNA contamination. Most enrichment protocols rely on alkaline lysis, which selectively denatures chromosomal DNA while allowing small, supercoiled plasmids to renature and remain soluble [27]. However, this approach preferentially recovers small plasmids and often leads to the loss of large plasmids during extraction [28], resulting in their underrepresentation despite their potential functional importance.

Other methods employ PlasmidSafe DNase to selectively digest linear dsDNA, preserving large circular plasmids [29, 30]. However, this strategy excludes linear plasmids from downstream analyses. Using this approach, Calderón-Osorio *et al*. [29] reported that only 8.2% of metagenomic reads from river samples were classified as plasmid-derived based on the plasmid RefSeq database. This low proportion suggests that a substantial fraction of plasmid diversity remains uncharacterized, likely due to biases towards human-associated bacteria in reference databases (e.g. RefSeq) and/or residual chromosomal contamination in plasmid-enriched fractions. Collectively, these findings underscore major limitations in current plasmid ecology studies and highlight the urgent need to expand plasmid reference databases to better represent environmental bacterial genomes and metagenomes [31, 32]. In this regard, relevant tools and databases for plasmidomic analyses are summarized in Table 1.

**Table 1.** Relevant tools and databases for plasmidomic analyses

Tool					
**Name**	Input data	Approach	Typical application	Main limitation	Reference
**PlasmidFinder**	Assembled contigs	Replicon-based identification using incompatibility group markers	Plasmid typing and incompatibility group assignment	- Limited sensitivity; detects only known replicons - Restricted to certain taxonomic groups (mainly Enterobacteriaceae and Gram-positives) - Poor performance on novel or highly fragmented plasmids - Limited for genome assemblies	[11]
**plasmidSPAdes**	Raw sequencing reads	Assembly graph-based reconstruction using coverage differences	*De novo* plasmid assembly from whole genome sequencing data	- Requires high and differential coverage - Poor resolution for low-copy plasmids with coverage similar to chromosome - Limited for genome assemblies	[51]
**MOB-suite**	Assembled contigs	Marker gene sequences+contig clustering using mash genomic distance+MOB typing	Plasmid reconstruction, relaxase/MOB typing, conjugation potential prediction and host range prediction	- Limited for genome assemblies - Dependent on assembly quality - Database-dependent for novel plasmids	[13, 52]
**PlasX**	Assembled metagenomic contigs	Machine learning (logistic regression) using *de novo* gene families (1,090,132 families); trained on 16,827 plasmids+14,367 chromosomes	Identification of plasmid-derived contigs in metagenomes	- Model-dependent - Classification based on an arbitrary score threshold - Cannot recognize plasmids with signatures not in training data - 81.5% recall on PLSDB plasmids	[10]
**PlasFlow**	Assembled metagenomic contigs (≥1 kb recommended)	Deep neural network using k-mer frequencies and genome signatures; ensemble voting classifier with probability threshold filtering trained on 7,604 plasmids+1,961 chromosomes	Identification of plasmid-derived contigs with phylum-level taxonomic classification (96% accuracy)	- No longer actively maintained (highly important) - Model dependent - Cannot recognize plasmids with signatures not in training data - 85.98% recall - May confuse integrated elements - Not recommended for short sequences	[16]
**Platon**	Assembled contigs	Replicon Distribution Score+plasmidic marker protein sequences+heuristics	Identification of plasmid-derived contigs in isolate genomes and metagenomes	- Model dependent - Cannot recognize plasmids with signatures not in training data - Moderate sensitivity (~70%) - Automatically classifies contigs >500 kbp as chromosomal - 37.4% recall on PLSDB plasmids	[14]
**geNomad**	Assembled contigs	A deep neural network integrating both an alignment-free classifier and a protein-based classifier trained on a custom marker dataset	Identification of plasmid-derived contigs in isolate genomes and metagenomes; also applicable to viral sequence identification	- 77.8% of classification performance on plasmids - Classification based on an arbitrary score threshold - Cannot recognize plasmids with signatures not in training data	[53]
**Databases**					
**PlasmidScope**	Plasmid sequences	Comprehensive reference database (852,600 plasmids from 10 repositories)+annotation pipeline	Plasmid annotation, classification, comparative analysis and online analytical tools	- Database tool (not *de novo* identification); plasmid identification must be done separately	[32]
**IMG/PR**	Plasmid sequences	Curated reference database with functional, taxonomic and ecological metadata (699,973 plasmids from genomes, metagenomes and metatranscriptomes)	Comparative plasmid genomics, plasmid taxonomic unit (PTU) definition, host range and functional profiling	- Database resource only (requires external tools for analysis); plasmid identification must be done separately	[31]
**PLSDB**	Plasmid sequences	Curated reference database derived from NCBI prokaryotic genomes (72,360 plasmids)	Plasmid comparison and annotation of complete plasmids; plasmid detection using genomic distances against the database	- Includes only complete plasmids from NCBI; plasmids derived from metagenomic datasets are underrepresented - Database dependent for novel plasmids	[54, 55]

These methodological constraints are not merely technical limitations; they directly determine the resolution at which plasmid ecology can be studied. Gaps in assembly continuity, database coverage and host assignment each introduce systematic blind spots in our understanding of how plasmid communities are structured across environments.

## The need for a consolidated plasmid classification system

Another major limitation for understanding plasmid ecological dynamics is the absence of a universal classification system. Existing frameworks based on host range or marker typing systematically fail to capture ecologically meaningful units, particularly given that a substantial proportion of environmental plasmids lack these canonical markers or cannot be identified and that host-range inferences are often constrained by the limited diversity of cultivar-derived reference databases. Although several classification approaches have been proposed (reviewed in [33] and summarized in Table 2), none has achieved the broad acceptance or level of standardization comparable to taxonomic systems developed for cellular organisms or, more recently, for viruses [34].

**Table 2.** Advantages and limitations of the main plasmid classification systems reviewed in [33]

Classification system	Key feature	Advantage	Limitation
**Fertility inhibition (fi**)	- Ability to inhibit F plasmid conjugative transfer; *fi+* plasmids inhibit F transfer, whereas *fi−* plasmids do not	- Early systematic approach; helped identify relationships between plasmids	- Applied to two different phenomena - Very limited scope; gradually abandoned
**Incompatibility**	- Stable coexistence in the same cell; shared replication and partition systems	- Broad consensus effort with several Inc groups established - Widely used in epidemiological studies - Constituted the basis for the replicon typing system	- Not testable in plasmids derived from unculturable MAGs - Plasmids may break incompatibility rules - Requires mating experiments to test plasmid stability - Requires different antibiotic markers - Exponentially increasing test combinations
**Replicon typing**	- Replication origin sequences - Initially based on DNA hybridization and PCR, later moved to genome sequence-based detection (PlasmidFinder) - Includes plasmid Multi Locus Sequence Type for some Inc groups - 471 replicon sequences	- Universal plasmid characteristics - Well-established databases	- Essentially a genetic analogue of the incompatibility system - Restricted to certain taxonomic groups (mainly Enterobacteriaceae and Gram-positives) - Multi-replicon plasmids cannot be classified into a single group - Same replicon in different PTUs - Cut-off values do not guarantee incompatibility - Dependent on known replicon sequences
**MOB class**	- Relaxase gene identity and phylogeny - Nine MOB classes - PCR typing available (for those in *Gammaproteobacteria*) - MOBscan web server [56]	- Robust universal marker - Only gene shared by most transmissible elements - More consistent than replicon typing - Smaller number of classes (9 vs 471 replicons)	- Cannot be applied to non-mobilizable plasmids which represent a great portion of plasmids - MOB and Rep phylogenies not always congruent - Single-gene limitations
**pATLAS [57]**	- Alignment-free (Mash, *k*=21) - Visual network tool including 13,924 plasmids Jaccard index threshold=0.9	- User-friendly exploration tool - Can identify plasmids similar to queries - Includes metadata	- No further community definition - No true classification - Not updated
**Plasmid cliques [58]**	- Alignment-free (BinDash, *k*=21) - 566 cliques (plasmid groups) with 561 containing 3 or more plasmids - Jaccard index threshold=0.3	- Highly correlated with gene content, host, G+C content, replicon type and MOB class - Can infer HGT events	- Some plasmids assigned to multiple cliques - Stringent threshold (low diversity) - No automatic allocation tool for new plasmid sequences
**MOB-suite clustering [13]**	- Alignment-free (Mash, *k*=21) - 7,298 clusters - Complete-linkage hierarchical clustering - Mash distance threshold=0.06	- Includes MOB-typer and MOB-recon tools - Plasmid reconstruction from draft assemblies - Automated classification tool available	- Replicon prediction does not totally agree with PlasmidFinder database - Stringent threshold leading to 53% singletons - Limited to Proteobacteria MOB/MPF types
**PTUs [35]**	- Alignment-based (ANIL50) - 380 PTUs - Hierarchical Bayesian stochastic block modelling-based algorithm using ≥70% sequence identity across ≥50% alignment coverage of the smaller genome	- No bias towards specific characteristics - Clear genetic discontinuity - Includes cliques and paracliques - COPLA: tool for automatic assignment (web-based) host-range classification	- 253/380 PTUs lack PlasmidFinder replicons - Members of the same PTU may be compatible (do not match Inc groups) - Plasmids within the same PTU can harbour distinct relaxase (MOB) classes - Purely threshold-based clustering with limited biological relevance - Studies linking PTU structure to biological or ecological traits remain scarce

The precedent of virus taxonomy constitutes a valuable guideline to consider. Despite not being considered living organisms, viruses have long benefited from a formal taxonomic framework (with the exception of most phages); and as the availability of viral genomes expanded exponentially (including from uncultured sources) the International Committee on Taxonomy of Viruses adopted the genome-based similarity thresholds, defining species at ~95% average nucleotide identity (ANI) and genera at ~70%. This transition, however, highlights the broader challenge of how taxonomic systems should be updated and on what criteria they should be based.

In plasmid biology, we are now facing an analogous inflection point. The rapid expansion of plasmid sequences has substantially broadened our understanding of their diversity and ecology, yet it also exposes the limitations of existing classification frameworks. This growing body of data demands a classification system grounded in genomic and ecological criteria, rather than relying on phenotypic traits or host-associated proxies. Accordingly, developing a consolidated, ecology-informed plasmid taxonomy is not an isolated methodological challenge but a direct consequence of recognizing plasmids as ecological entities – the central argument advanced throughout this perspective.

A more recent genome-based strategy was explored by Redondo-Salvo *et al*. [35], who clustered more than 10,000 plasmids into plasmid taxonomic units (PTUs) based on high sequence similarity. PTU reconstruction relied on an alignment fraction (AF) of ≥50%, which is defined as the percentage of two DNA molecules that can be unambiguously aligned above a given similarity threshold, and an ANI threshold of 70%. This combination was designed to avoid spurious clustering of short plasmid sequences sharing only transposable elements. This approach was later adopted by the Integrated Microbial Genome (IMG) team, which applied a similar approach for constructing their plasmid database (Table 1) [31].

However, clustering-based approaches can vary substantially depending on the software and algorithms used to group plasmid sequences, even when applying the same parameters. This issue is particularly pronounced when clustering large graphs, where small changes in network structure can lead to markedly different cluster definitions. For example, the IMG team employed vclust with the Leiden algorithm to define PTUs using the parameters described above [31]. However, vclust implements multiple clustering algorithms, each of which can produce distinct plasmid groupings [36]. Similar limitations apply to other tools for plasmid classification which are based on clustering algorithms. Importantly, the choice of clustering approach should be guided by the specific scientific objective. Although most of these methods do not provide a formal taxonomic framework, they can still be valuable for addressing ecological questions related to plasmid diversity and structure.

The main advantage of the PTU-based approaches is its reliance on whole-genome sequences for clustering. By using complete sequence information, it captures a broader spectrum of plasmid features and allows the inclusion of plasmids that lack canonical mobilization genes commonly used for classification, or even the absence of replication proteins (for plasmid fragments or with unknown replicases). And despite the ANI-based tools do not infer phylogenetic relationships by modelling the evolutionary trajectory of sequences, in bacterial genomes such distances may strongly correlate with phylogenetic analyses, often reflecting evolutionary classifications [37].

Nevertheless, this strategy remains constrained by important limitations. For example, in the IMG/PR analysis, 699,973 plasmid sequences were clustered into 214,950 PTUs, with ~63% represented by a single plasmid. This high proportion of singletons indicates that even whole-genome-based approaches may fail to fully reconstruct plasmid evolutionary relationships [31]. However, it is important to consider that the IMG/PR database includes several sequences classified as plasmids from metagenomic datasets, which may represent false positives or fragmented plasmid sequences, thereby introducing additional noise into downstream analyses.

Moreover, plasmids evolve in a highly modular manner, undergo frequent recombination, and can change rapidly over short evolutionary timescales, thereby readily disrupting fixed identity and AF thresholds used for clustering. More fundamentally, this limitation echoes an assumption previously raised by Garcillán-Barcia *et al*. [1]: when a plasmid has gradually replaced its entire original gene repertoire, does it retain its identity, or has it effectively become a different entity? This ‘plasmid Theseus’ paradox’ highlights the conceptual difficulty of reconstructing plasmid macro-evolutionary lineages that allow to identify plasmids originated from a common ancestor even when they are now considered distinct entities; and suggests intrinsic limits to any clustering method lacking phylogenetic analysis for plasmids. Collectively, these observations underscore the need for future studies to move beyond static bioinformatic classification schemes and instead explicitly incorporate the ecological and evolutionary processes shaping plasmid diversity and modularity.

An alternative approach that can capture the gradual functional changes of plasmids is the use of best bidirectional hits (BBHs) between gene sequences, particularly focusing on proteins that tend to be more conserved. This strategy has been widely applied to cluster many different molecular symbionts such as plasmids, ICEs and phage–plasmids [20, 38]. By emphasizing shared protein content, especially those forming the plasmid ‘backbone’ (i.e. genes involved in replication, maintenance and transfer [10, 39]), it becomes possible to map relationships between simpler backbone plasmids and more complex derivatives carrying additional cargo genes, as implemented by Yu *et al*. in the MobMess tool [10].

By relying on shared protein content rather than strict ANI-based thresholds, protein-based approaches can recover deeper and more divergent evolutionary relationships among molecular symbionts that retain at least a minimal set of homologous proteins. This enables the detection of large-scale evolutionary patterns that are often obscured by nucleotide identity-based methods. However, even when a set of plasmids derive from a common ancestor and still share a minimal set of proteins, subsequent diversification (through HGT, recombination or plasmid fusion events) can erode this shared backbone to the point that common ancestry is no longer readily detectable by sequence similarity or clustering approaches. In such cases, phylogenetic analyses based on carefully selected marker genes may provide additional resolution, although these too are constrained by the inherently mosaic nature of plasmids.

Therefore, protein- or gene-content-based frameworks may offer a more biologically meaningful basis for plasmid classification. One promising strategy is a hierarchical approach in which plasmids are first grouped according to phylogenetic relationships of key functional modules – such as replication systems (Inc groups), mobility determinants (e.g. relaxases), and conjugation machinery (secretion systems) – and are then further refined based on conserved gene content within the plasmid backbone or ANI-based methods.

Despite these advantages, BBH-based clustering has not yet been systematically applied to develop a plasmid classification system using a comprehensive plasmid dataset, representing a critical gap in current plasmid research. A key limitation is the requirement for accurate annotation of coding sequences, a computational and tool-dependent step that may introduce variability across analyses. Nevertheless, integrating protein- or gene-content-based and ANI-based approaches together may provide a more robust framework for resolving plasmid evolutionary relationships and advancing towards a biologically grounded plasmid classification system.

The limitations outlined above extend beyond methodological limits and point to a more fundamental issue in how plasmids are studied and classified. Recent plasmidomic studies have begun to address this gap, revealing ecological patterns that challenge traditional host-centric views of plasmid biology. Importantly, the findings discussed in the following section should be interpreted as conservative estimates of the true complexity of plasmid ecology, still constrained by the methodological and taxonomic limitations described above, yet already compelling enough to underscore the need for a more systematic and ecology-informed exploration of plasmid dynamics.

## Plasmid ecology goes beyond host ecology

One major limitation of classifying or studying plasmids based on host taxonomy is that plasmids can exhibit ecological patterns that are decoupled from their hosts as previously discussed. For example, plasmids derived from the human gut microbiomes of individuals from industrialized countries have been reported to form distinct plasmid systems compared with those from non-industrialized countries [10], with variable gene repertoires, including genes related to antibiotic resistance and efflux pumps. Notably, most predicted plasmids in that study did not display distributions correlated with their putative host taxonomy, highlighting plasmids as highly dynamic components of microbiomes with ecological patterns different from those of their bacterial hosts (Fig. 1a). Although the approach used by the authors to estimate host-taxonomy represents a more rigorous mathematical framework – leveraging Pearson correlations and Jaccard indices to estimate ecological co-occurrence between plasmids and potential hosts across metagenomes – it remains dependent on short-read data and read mapping-based taxonomic assignment, inheriting the assembly fragmentation and databases-detection biases outlined above.

**Fig. 1.**
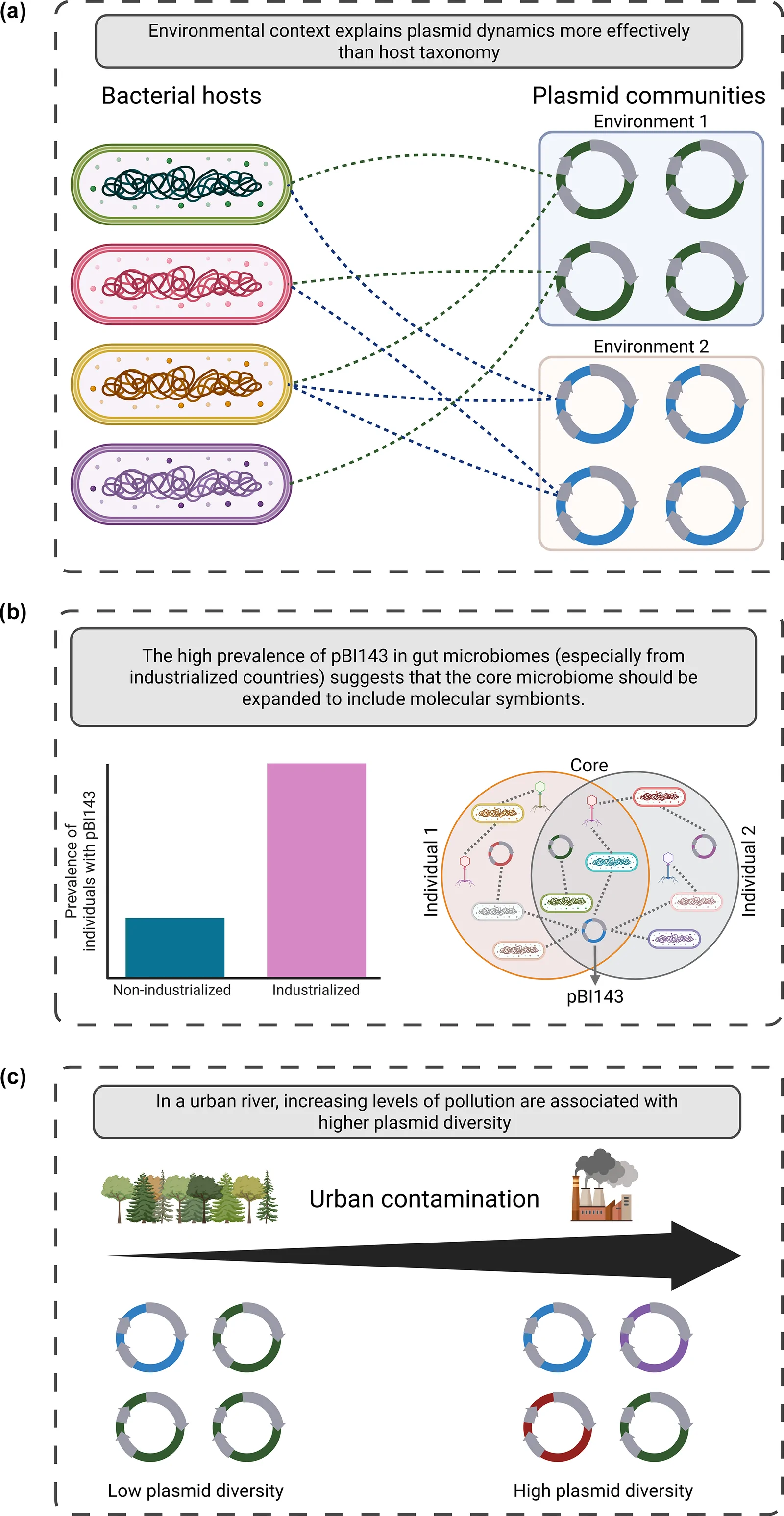
Some examples of how plasmid ecology goes beyond host taxonomy. (**a**) Plasmid communities are structured more strongly by environment than by host taxonomy. (**b**) The prevalence of pBI143, one of the most abundant genetic elements in the human gut, is higher in industrialized countries, underscoring the need to expand the ‘core microbiome’ concept to include molecular symbionts. The grey dashed lines indicate molecular symbiont–host interactions, illustrating that some elements, such as pBI143, can interact with multiple hosts, while the hosts themselves may not belong to the core microbiome, the plasmid does. (**c**) Plasmid diversity increases with pollution levels.

Further illustrating this disconnect, the cryptic plasmid pBI143 appears to be one of the most abundant genetic elements in the human gut, predominantly detected in individuals from industrialized countries compared with those from non-industrialized countries [40]. Despite having no measurable impact on host fitness *in vivo*, its presence has been associated with inflammatory states in the human colon, underscoring the importance of plasmid dynamics in microbiome function. In that study, the authors raise the question of whether current and future research should expand the concept of a ‘core microbiome’ beyond microbial taxa to include plasmids and other molecular symbionts that are more prevalent than their hosts across populations, potentially advancing our understanding of microbial ecology (Fig. 1b). This is a compelling reframing with broad implications for microbiome research. Resolving it, however, requires methods capable of directly linking plasmids (and other molecular symbionts) to their hosts within complex communities, such as Hi-C metagenomics or meta-epigenomics (discussed further below).

Similarly, studies have shown that in an urban river, increasing levels of pollution are associated with higher plasmid diversity, while chromosomal diversity is reduced or remains stable [29, 41]. This pattern suggests that plasmid communities may respond more rapidly or sensitively to environmental stressors, consistent with the well-established role of plasmids as vectors of adaptive traits shaped by strong selective pressures (Fig. 1c). However, these analyses rely on short-read data mapped to plasmid databases derived from known hosts and use bacterial host taxonomy to interpret plasmid diversity, which may not accurately reflect the true diversity of plasmids or their associated hosts.

More broadly, inferring plasmid host taxonomy based solely on previously reported associations is inherently limited. While some plasmid lineages appear restricted to specific bacterial taxa, others exhibit broad host ranges that span distantly related species, including different classes or phyla [35, 42, 43]. Consequently, the true extent of plasmid host range in natural communities remains uncertain. Plasmid groups associated with diverse taxa through bioinformatic approaches may, in reality, successfully establish in only a limited number of hosts after overcoming multiple biological barriers. Conversely, the host range of other plasmids may be underestimated due to methodological limitations. In both cases, these uncertainties underscore the need for improved approaches that accurately resolve plasmid–host interactions in natural environments.

Some emerging approaches, such as Hi-C metagenomic sequencing or meta-epigenomics, offer promising alternatives and have been successfully applied to resolve molecular symbionts–host interactions [44–47]. In Hi-C metagenomics, shotgun sequencing is combined with high-throughput chromosome conformation capture, which provides proximity ligation information. This enables the study of complex microbial communities by identifying DNA fragments that are physically close or co-localized within the same cell. Such information is particularly valuable for linking contigs to their originating genomes and for identifying extrachromosomal elements (e.g., plasmids) associated with specific host cells, as previously reported [44, 45].

Similarly, other approaches have leveraged epigenetic signatures, such as DNA methylation, to improve metagenomic binning and to infer host–mobile element associations. For instance, Beaulaurier *et al*. [47] reconstructed poorly characterized genomes from mouse gut microbiomes and linked MGEs to their hosts based on shared methylation profiles obtained through Single Molecule Real-Time sequencing (PacBio). More recently, nanopore-based metagenomic sequencing has enabled the resolution of plasmid–host associations by exploiting methylation similarity across sequences, clustering contigs based on shared motif-level methylation patterns [46].

Although these approaches can substantially improve the resolution of host–plasmid associations in metagenomic data, they remain under active development and are subject to important limitations. In Hi-C metagenomics, smaller contigs with weak interaction signals tend to exhibit higher variance, which can compromise normalization procedures and reduce clustering stability when inferring contig–contig associations [48]. Similarly, meta-epigenomic approaches can be constrained in high-complexity samples (e.g. gut or environmental metagenomes), where reduced methylome uniqueness (due to the recurrence of shared methylation motifs across taxa) can hinder accurate clustering [47].

In addition, both approaches can be cost-prohibitive, limiting their widespread application. Consequently, most studies continue to rely primarily on short-read metagenomics. Accordingly, alternative tools have been developed to infer plasmid host taxonomy in short-read datasets using MLB [49, 50], but these methods also face biased limitations, as they depend on reference datasets of known plasmid–host associations.

## Concluding remarks and future perspectives

Plasmidomics is consolidating the view of plasmids as ecological entities whose diversity and evolutionary dynamics are shaped as much by environmental and intracellular contexts as by the population dynamics of their bacterial hosts. The evidence reviewed here consistently challenges host-centric frameworks: plasmid communities can respond independently to environmental gradients, with patterns that are not reflected in host taxonomy, highlighting the pervasive and distinct ecological roles of plasmids across ecosystems, including the human microbiome. Consequently, studying plasmids in isolation from their ecological context is no longer tenable, and future plasmidomic research must adopt integrative, ecology-informed frameworks.

Yet, the field faces compounding limitations that constrain this perception. Short-read assemblies and culture-dependent approaches may underrepresent (and overrepresent) environmental plasmid diversity and host range, meaning that current ecological patterns likely capture only a fraction of the true plasmid landscape. At the same time, the lack of a universal, ecology-informed classification system continues to hinder meaningful comparisons across studies and ecosystems. The integration of long-read sequencing, particularly when combined with Hi-C proximity ligation or meta-epigenomics, will be critical to resolve plasmid structure and host associations at scale. Finally, recognizing plasmids as ecological units calls for a reassessment of foundational concepts such as the ‘core microbiome’, expanding it to include persistent MGEs alongside their microbial hosts.

## Abbreviations

AF: alignment fraction
ANI: average nucleotide identity
BBH: best bidirectional hit
HGT: horizontal gene transfer
ICE: integrative and conjugative element
IMG: Integrated Microbial Genome
MAG: metagenome-assembled genome
MGE: mobile genetic element
MLB: machine learning based
PTU: plasmid taxonomic unit
